# The different ways to chitosan/hyaluronic acid nanoparticles: templated vs direct complexation. Influence of particle preparation on morphology, cell uptake and silencing efficiency

**DOI:** 10.3762/bjnano.10.250

**Published:** 2019-12-30

**Authors:** Arianna Gennari, Julio M Rios de la Rosa, Erwin Hohn, Maria Pelliccia, Enrique Lallana, Roberto Donno, Annalisa Tirella, Nicola Tirelli

**Affiliations:** 1 Laboratory of Polymers and Biomaterials, Fondazione Istituto Italiano di Tecnologia, 16163 Genova, Italy; 2 NorthWest Centre for Advanced Drug Delivery (NoWCADD), School of Health Sciences, University of Manchester, Oxford Road, Manchester, M13 9PT, United Kingdom; 3 Division of Pharmacy and Optometry, Faculty of Biology, Medicine and Health, Stopford Building, University of Manchester and Manchester Academic Health Science Centre, Manchester, M13 9PT, United Kingdom; 4 current address: Cambridge Enterprise Limited, University of Cambridge, Hauser Forum, 3 Charles Babbage Rd, Cambridge, CB3 0GT, United Kingdom; 5 current address: Novartis EBEWE Pharma Ges.m.b.H. Nfg.KG, Lehenau 10a, 5325 Plainfield, Austria; 6 current address: Orchard Therapeutics plc., 108 Cannon Street, EC4N 6EU London, United Kingdom

**Keywords:** aggregation, chitosan, field flow fractionation, light scattering, targeted drug delivery

## Abstract

This study is about linking preparative processes of nanoparticles with the morphology of the nanoparticles and with their efficiency in delivering payloads intracellularly. The nanoparticles are composed of hyaluronic acid (HA) and chitosan; the former can address a nanoparticle to cell surface receptors such as CD44, the second allows both for entrapment of nucleic acids and for an endosomolytic activity that facilitates their liberation in the cytoplasm. Here, we have systematically compared nanoparticles prepared either A) through a two-step process based on intermediate (template) particles produced via ionotropic gelation of chitosan with triphosphate (TPP), which are then incubated with HA, or B) through direct polyelectrolyte complexation of chitosan and HA. Here we demonstrate that HA is capable to quantitatively replace TPP in the template process and significant aggregation takes place during the TPP–HA exchange. The templated chitosan/HA nanoparticles therefore have a mildly larger size (measured by dynamic light scattering alone or by field flow fractionation coupled to static or dynamic light scattering), and above all a higher aspect ratio (*R*_g_/*R*_H_) and a lower fractal dimension. We then compared the kinetics of uptake and the (antiluciferase) siRNA delivery performance in murine RAW 264.7 macrophages and in human HCT-116 colorectal tumor cells. The preparative method (and therefore the internal particle morphology) had little effect on the uptake kinetics and no statistically relevant influence on silencing (templated particles often showing a lower silencing). Cell-specific factors, on the contrary, overwhelmingly determined the efficacy of the carriers, with, e.g., those containing low-MW chitosan performing better in macrophages and those with high-MW chitosan in HCT-116.

## Introduction

Chitosan is a linear copolymer of β-1,4-ᴅ-glucose-2-amine and *N*-acetyl-ᴅ-glucose-2-amine, and is commonly employed as the cationic component in polyplexes and other drug delivery vehicles [1–3]. In comparison to other polycations, its main advantages are the low toxicity and its biodegradability. Biodegradation can occur both enzymatically and oxidatively [4]. A number of methods can be employed to prepare chitosan-based nanoparticles [5–8], the most popular being ionotropic gelation and polyelectrolyte complexation. The distinction between the two is subtle, since they are based on a common driving force, i.e., the electrostatic attraction between protonated amines on chitosan and multiply charged anions, which effectively act as cross-linkers. Electrostatic complexation allows for very mild preparative processes, carried out in water under mild and almost physiological conditions and without the use of chemical reactions. Additionally, electrostatic interactions may not only be used to hold together a particle, but also to encapsulate and retain payloads such as nucleic acids (either in combination with other anionic components [9], or as the only negatively charged molecule [10]).

Ionotropic gelation and polyelectrolyte complexation use anionic components of different size, In the former, typically an inorganic anion of low molecular weight, such as triphosphate (TPP) is used, while in the latter negatively charged polymers, e.g., hyaluronic acid (HA) [11–12] or alginate [13], are commonly used. HA is a particularly interesting component, since its presence allows for a reduced serum protein adsorption on chitosan-containing nanoparticles [14] and a receptor-mediated mechanism of internalization [15]. A larger size of the anionic component corresponds to a higher avidity toward chitosan, thus polyelectrolyte complexes are more stable but also difficult to reverse; this irreversibility makes polyelectrolyte complexation largely a kinetically controlled process, the details of which are in principle more difficult to reproduce. Roughly half way between the two processes, in several cases a low-MW polyanion is used together with a macromolecular anion, attempting to combine stability and reversibility; this is the case with TPP and HA, which have been used together in one-pot preparations [11], or in sequence, i.e., first producing chitosan/TPP nanoparticles, and then adding HA [16–17]. In the latter process, we noticed that molecular weight of chitosan influenced the presentation of HA [18], which affected the nanoparticle internalization in both RAW 264.7 macrophages [19–20] and XS106 dendritic cells [21]. In both cases, nanoparticles based on chitosan of low molecular weight appeared to be surrounded by a corona of loosely bound HA, which on one hand lowered the maximum amount of internalizable particles, but on the other hand made it more sensitive to the presence of additional biofunctional groups on HA [21].

There are, however, several yet unanswered questions regarding the use of TPP in the preparation of nanoparticles, when they also contain HA. These questions are, in particular:

1. whether and how much TPP is retained in the particles, since chitosan–TPP interactions are more easily reversed than chitosan–HA interactions (lower avidity);
2. whether the initial presence of TPP makes the final particles morphologically different; in previous reports, we have referred to the product of the two-step complexation (first between chitosan and TPP, then addition of HA) as HA-coated nanoparticles. Actually, HA is used as a last step and in excess, thus it ought to be the dominant component on the surface of the polyelectrolyte complex. However, the definition of HA-coated particles implies a yet-to-be-proven core–shell structure, as opposed to homogeneous particles obtained via direct chitosan/HA complexation;
3. whether the different process, and the possibly associated differences in composition and morphology may result in a biologically different performance in the cellular delivery of a payload.

**Scheme 1:**
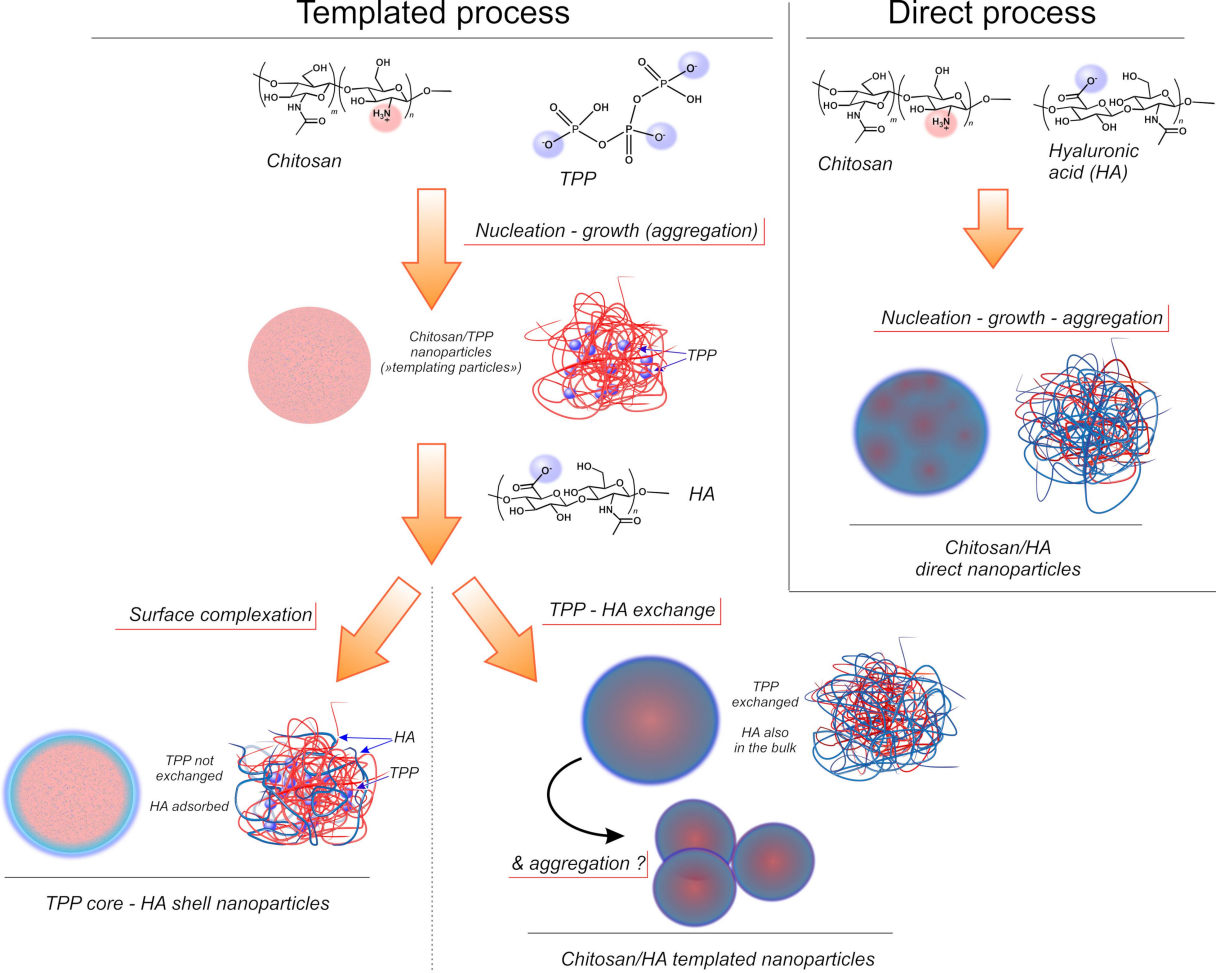
In the templated process (left), in a lightly acidic aqueous solution the protonated chitosan amines (cationic species in red) are cross-linked by TPP (anionic species in blue). The complexation leads to the nucleation of the particles that increase in size likely more through the adsorption of additional chitosan and TPP species (growth) than through aggregation of smaller intermediate particles, as the ζ-potential of the nanoparticles supposedly always remains positive, and therefore repulsive. When exposed to the polyanionic HA, the two limiting conditions are that the templating nanoparticles either A) adsorb it on their cationic surface, inverting their ζ-potential and therefore yielding HA-coated, core–shell nanoparticles (bottom left), or B) allow HA to replace TPP in their bulk. The latter process likely is morphologically templated, since it occurs within the nanoparticles. In a process based on the direct complexation between chitosan and HA (right), nanoparticles nucleate and then increase in size via either adsorption of individual components (growth) or via aggregation due the presence of patches of different charge on their surface. The possibility of aggregation may lead to a more heterogeneous bulk structure than in the templated process.

Here, we have compared the physico-chemical and carrier properties of the product of chitosan/HA polyelectrolyte complexation (“direct process” in Scheme 1) and that of the process where chitosan–TPP nanoparticles are produced first and act as a kind of template for the final material (“templated process” in Scheme 1). For the latter process, we also aimed to elucidate whether the HA is incorporated in the particles either via surface adsorption or bulk reorganization as showed in Scheme 1.

We then assessed how the possible morphological differences introduced by the different preparative processes may affect nanoparticle uptake and the release of encapsulated payloads using a murine macrophagic (RAW 264.7) and a human colorectal tumor (HCT-116) cell line, both popular in vitro models for the study of cellular interactions of HA-based materials [20,22].

## Experimental

### Materials and methods

The list of chemicals used is provided in Supporting Information File 1, Section SI1.1. Chitosan of viscosity average molecular weight 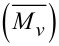 656 kDa and a degree of deacetylation (DD) of 85% (from ^1^H NMR; hereafter referred to as Chit_650_ or high-MW chitosan) was purchased from Sigma-Aldrich (Gillingham, UK) and purified prior to use as previously described (boiling in 2% acetic acid, filtration, precipitation with NaOH, ultrafiltration and freeze drying) [16]. Chitosan with 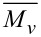 = 36 kDa and a DD = 85% (hereafter referred to as Chit_35_ or low-MW chitosan) was obtained by oxidative degradation of the above high-MW chitosan (1 wt % in 0.1 M HCl/3 mM sodium nitrite, room temperature, 12 h) [21]. Hyaluronic acid (HA) with weight average molecular weight 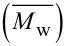 = 180 kDa was kindly donated by Kyowa Hakko Bio Italia Srl (Milan, Italy).

### Preparation of chitosan/HA nanoparticles

In all cases, the nanoparticles were prepared under sterile conditions (Cat. II cabinet) with surfaces previously treated with RNaseZap^®^ solution (Thermo Scientific, UK) for the handling of nucleic acids. All the steps were performed under vigorous magnetic stirring (1,000 rpm) and at 25 °C in 2 mL round bottom Eppendorf tubes.

**A) Template-based (TPP) method.** In a typical experiment, nanoparticles were prepared by the addition of 72 µL of a 0.1 wt % solution of TPP in deionized water (pH 5 adjusted with 0.1 M HCl) to 928 µL of a 0.069 wt % solution of Chit_35_ or Chit_650_ in 4.6 mM HCl (pH 5 adjusted with 0.1 M NaOH), always at a 1:9 TPP/chitosan weight ratio. In the case of siRNA-loaded nanoparticles, TPP was dissolved in deionized nuclease-free water containing siRNA. The chitosan–TPP nanoparticle (template) dispersion was stirred for 30 min and then mixed with 1 mL of acetate buffer (200 mM, pH 5). After 5 min, 1 mL of the nanoparticle suspension was added to an equal volume of a 1.5 mg/mL HA in acetate buffer (100 mM, pH 5) and kept under stirring for 30 min, always at 25 °C. The resulting nanoparticle (chitosan–TPP/HA) suspension was then dialyzed against deionized water (MWCO = 1,000 kDa) for 5 h, changing the water every 20 min.

**B) Direct complexation.** 500 µL of a 0.069 wt % chitosan solution prepared as described above was mixed with an equal volume of deionized and nuclease-free water containing siRNA or simply with deionized water for 10 min at 25 °C. The resulting dispersion (with or without siRNA) was added to 1 mL of a 1.5 mg/mL HA solution in water (previously adjusted to pH 5) and stirred for 30 min.

Fluorescently labelled particles were produced using chitosan and HA, which were appropriately labelled as described in Supporting Information File 1, Section SI1.2.

### Nanoparticle characterization

**Elemental analysis.** 5 mL of nanoparticle suspension were dialyzed against deionized water (MWCO = 1,000 kDa) for 5 h, changing the water every 20 min. The composition of freeze-dried nanoparticles was then analysed using a Thermo Flash 2000 Organic Elemental Analyser for carbon (C) and nitrogen (N), and a Thermo Scientific iCAP 6300 DUO ICP Spectrometer for phosphorus (P). Theoretical compositions were calculated under assumption that: i) all glucosamine units in chitosan are protonated complexed to either TPP or HA; ii) TPP has an average of three negative charges (at pH 5.0, which means that two oxygens are still protonated); iii) HA is completely deprotonated and all its carboxylates are either complexed with chitosan or present as sodium salt.

**Hydrodynamic size and ζ-potential.** Z-average hydrodynamic size, polydispersity index (PDI), and ζ-potential were measured on three independent samples at 25 °C using a Zetasizer Nano ZS instrument (Model ZEN3600, Malvern Instruments Ltd., UK) equipped with a solid state HeNe laser (λ = 633 nm) at a scattering angle of 173°. Size measurement data were obtained by using the General Purpose algorithm. The electrophoretic mobility of nanoparticles was converted into ζ-potential values by means of the Smoluchowski equation using Malvern Zetasizer software (v7.11).

**Capillary electrophoresis.** Electrophoresis measurements were performed at 25 °C on a P/ACE MDQ Plus (SCIEX, Warrington, UK) equipped with a 50 cm effective length (70 cm total length) capillary with 75 μm internal diameter (Beckam Coulter, Brea, USA) and a photodiode array detector operating at 214 nm. The capillary was first conditioned by successive rinsing steps (all performed at 20 psi): 5 min with 1 M HCl, 2 min with deionized water, 10 min with 0.1 M NaOH, 2 min with deionized water, 5 min with 20 mM phosphate buffer at pH 7.4 (running buffer), 1 min with 0.1 M NaOH, 1 min with deionized water, and finally 1.5 min with the running buffer. Each sample was then injected at 0.5 psi for 10 s, applying 15 kV between the anode and the injection site (normal polarity) for 60 min. At the end of each measurement, the capillary was rinsed with water for 1 min at 20 psi. Data acquisition and analysis were performed respectively with software packages 32 Karat (SCIEX) and OriginPro 8.5.1 (OriginLab Corporation, US). A calibration curve was obtained by injecting HA at known concentration (47, 188 and 750 g/mL), thus allowing to quantify the amount of unbound HA from the area of its peak at 22 min.

**Asymmetric-flow field flow fractionation (AF4).** An AF2000 TM (Postnova Analytics, Landsberg, Germany) featuring an A4F channel an equipped with a 350 µm spacer and a regenerated cellulose 10 kDa MWCO membrane as accumulation wall was employed in connection with a UV–vis detector operating at 220 nm (S3210, Laserchrom, Rochester, UK), a MALS detector (Viscotek SEC-MALS20, Malvern Instruments, Worcestershire, UK), a refractive index detector (Optilab T-rEX, Wyatt Technology, Dernbach, Germany) and a DLS (Zetasizer Nano SZ, Malvern) in the given order. A 0.02% (w/v) NaN_3_ solution filtered through a 0.1 µm pore size filter was used as the eluent. Prior to injection, the nanoparticle suspensions were concentrated to 3 mg/mL via ultrafiltration by using a membrane with MWCO of 10 kDa and DLS was performed to check that no aggregation occurred during this step. In a typical experiment, parameters were set as: 1) the detector flow rate 0.5 mL/min, 2) 100 µL of samples injected over 10 min at 0.3 mL/min, 3) cross flow rate 2.0 mL/min, and 4) focusing flow rate 2.70 mL/min (focusing step). During the elution step, the cross flow was kept constant at 2.0 mL/min for 0.5 min and then exponentially decreased (exponent = 0.40) to 0.09 mL/min over 30 min, and further exponentially decreased (exponent = 0.90) to 0.07 mL/min over 7 min, and kept constant at this value (0.07 mL/min) for 20 min. A rinse step was finally performed for 2 min, i.e., setting cross flow at 0 mL/min and purge valve on. UV–vis, MALS and refractive index data were analysed using AF2000 software (Postnova Analytics GmbH, Germany) and fitted with a Sphere model to obtain the MW and radius of gyration (*R*_g_) distributions. DLS data were analysed using the Zetasizer Nano software (Malvern). The data were also used to calculate:

1. ρ = *R*_g_/*R*_h_ (shape factor). This parameter defines key geometrical characteristics of a colloid. ρ values are given in literature [23] for a variety of particle morphologies. For example, ρ = 0.775 for a hard, uniform sphere, 1.0 for vesicles with thin walls (hollow spheres), close to 1.5 for random polymer coil conformations [23–24].
2. Fractal dimension (*D*). When applied to particulates, the fractal geometry analysis is another important morphological indicator. For example, aggregation of colloidal suspensions typically produces objects for which the mass can be expressed as fractal power of the size (mass fractals [25–26]), i.e., 
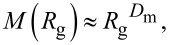
 where *D*_m_ (≤3) is the so-called mass fractal dimension of the particle aggregate system [27]. For instance, this parameter takes values greater than 2.5 for densely packed particle aggregates, whereas lower *D*_m_ values have been ascribed to more branched structures. Mass fractal dimensions of selected topologies have been calculated and can be found elsewhere [23]. In static light scattering, the expression is often approximated with the angular dependency of scattered intensity 
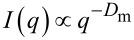
 expressed as a function of the scattering vector *q* = (4π*n*/λ)·sin(Θ/2) but here it was directly obtained as the slope of the nanoparticle mass vs *R*_g_ log–log plot.

**siRNA loading.** Encapsulation efficiency (EE) values (%) were calculated as EE (*A* – *B*)/*A*·100% where *A* is the amount of siRNA in the feed and *B* is the amount of non-complexed siRNA. The latter was quantified by separating as-prepared nanoparticles from the supernatant through centrifugation at 13,000 rpm for 60 min, and detecting siRNA in the supernatant with RiboGreen^®^ following the manufacturer’s instructions and using a Synergy2 Biotek plate reader.

**Atomic force microscopy (AFM).** Drops (ca. 35 µL) of the chitosan/HA nanoparticle suspensions were deposited on a clean mica surface and left to dry overnight in Petri dishes at room temperature. A molecular force probe 3D AFM (MFP-3D, Asylum Research, Oxford Instruments, Abingdon, UK) equipped with an OTESPA-R3 cantilever (Bruker, Camarillo, CA, USA) was used to acquire AFM images in air at room temperature in tapping mode. Igor-Pro AFM software (Oxford Instrument, UK) was used to analyse the images.

**Nuclease protection assay.** The protection effect against nuclease degradation of low-*M*_W_ and high-*M*_W_ chitosan nanoparticles prepared by ionotropic gelation or direct polyelectrolyte complexation was assayed by gel electrophoresis after nuclease and chitosanase/heparin treatment, as already described in [9]. Briefly, 44 µL of siRNA either loaded in the nanoparticles (25 wt % loading with respect to chitosan, a high siRNA loading for precise gel analysis) or dissolved in water at the same concentration (40 µg/mL) were incubated with 22 µL of a solution of RNAse I (15 mM Tris buffer, 0.3 M NaCl, pH 7.0) at a concentration of 0, 0.33 and 3.33 U (corresponding to 0, 0.5, and 5 U of enzymes per 100 µL of final reaction volume, respectively) at 37 °C for 30 min. The nuclease reaction was then quenched with the addition of 7.6 µL of 1.0% SDS (aq). Afterwards, 3 µL of a 0.084 units/µL solution of chitosanase in 50 mM acetate buffer (pH 5.0) were added to the mixture, and the enzymatic reaction was allowed to occur for 3 h. Finally, 4.7 µL of a solution of heparin (200 mg/mL in nuclease-free water; corresponding approximately to a z-Hep/z-siRNA ratio of 250) were added. The resulting mixture was incubated overnight at 25 °C. After centrifugation (13,000 rpm, 30 min), the nucleic acid released in solution was quantified using polyacrylamide gel electrophoresis (PAGE, 18-well/30 µL, 15% Criterion TM TBE-Urea Gel, Biorad; 70 min, 120 V). Gels were imaged with a UV trans-illuminator (ChemiDoc™ MP System #170-8280) adjusting the exposure time to avoid saturation, and the acquired images were analysed using ImageJ software (v1.49p, http://rsb.info.nih.gov/ij).

### Cell studies

HCT-116 and RAW 264.7 cell lines were cultured in complete media (McCoy’s 5A or high glucose DMEM, respectively) under standard conditions for cell culture (5% v/v CO_2_ in air, 37 °C). Further details of the materials used is provided in Supporting Information File 1, Section SI1.1.2.

**Preparation of double-concentrated cell culture growth media.** 5.95 g McCoy’s 5A powder or 6.75 g of DMEM powder, respectively, were dissolved in 175 mL of distilled water followed by addition of 3 g of HEPES. The pH value was then adjusted to 7.4 by adding 1 M HCl and the volume brought to 195 mL with distilled water. The resulting medium was filtered using disposable sterile filter systems (0.22 μm) and supplemented accordingly (20% v/v FBS, 2% v/v Penicilin-Streptomycin), referred to a final volume of 250 mL. Nanoparticle solutions for cellular experiments were prepared by the addition of a given volume of double-concentrated full growth medium to an equal volume of double-concentrated nanoparticle dispersion (water).

**Cytotoxicity experiments.** HCT-116 (20,000 cells/cm^2^) and RAW 264.7 (30,000 cells/cm^2^) were seeded in 48-well plates and left to adhere overnight (5% v/v CO_2_ in air, 37 °C). Cells were then exposed to 0.25 mL of nanoparticle suspensions in full medium (concentration: 0.01–0.5 mg/mL) for 24 h, then determining viability using the CellTiter 96^®^ AQueous One Solution Cell Proliferation Assay (MTS assay). Briefly, cells were washed with PBS and incubated for 1 h at 37 °C in medium containing 5% (v/v) of MTS solution. Cell viability was measured by reading the absorbance values at 490 nm (Synergy2 Biotek plate reader using Gen5 software) and normalized against the total protein content in each well (BCA assay). Please note that any influence of phenol-red was ruled out by using medium as blank and subtracting its absorbance to all wells before calculating metabolic activity.

**Quantification of cell uptake.** HCT-116 (20,000 cells/cm^2^) and RAW 264.7 (30,000 cells/cm^2^) were seeded in 12-well plates and left to adhere overnight (5% v/v CO_2_ in air, 37 °C). Cells were then incubated with 1 mL of fluorescently-labelled nanoparticles (particles produced with either RITC-labelled chitosan or rhodamine-labelled HA; for their synthesis see Supporting Information File 1, Section SI1.2) diluted to 125 µg/mL at 37 °C for specified incubation times, i.e., 0, 2, 4, 8, 16, and 24 h. Afterwards, cells were washed three times with pre-warmed PBS and lysed in 100 µL RIPA Buffer. The total uptake (combined membrane-bound and internalized materials) was calculated from fluorescence measurements of the cell lysates using a calibration curve from nanoparticle aqueous suspensions diluted in cell lysates (range 0.12–125 µg/mL). Measurements were obtained by using a Synergy2 Biotek plate reader (Ex 540/25, Em 620/40 nm), Gen5 software; top 50% optical position. Uptake results were normalized against the total protein content per well (BCA assay).

**Silencing experiments.** HCT-116 (20,000 cells/cm^2^) and RAW 264.7 (30,000 cells/cm^2^) were seeded in 48-well plates and left to adhere overnight (5% v/v CO_2_ in air, 37 °C). Cells were pre-transfected for 4 h with 0.25 µg of pGL3 vector encapsulated in Lipofectamine™ LTX according to manufacturer’s instructions. After subsequent removal of medium and gentle washing with warmed PBS, 0.25 mL of complete medium containing 0.67 µg of anti-Luc siRNA (200 nM) encapsulated in nanoparticles (125 µg/mL) were added to each well, with anti-Luc siRNA/LTX complex used as a positive control for transfection. Cells were incubated for 4 h with the nanoparticles, then medium was discarded and cells were washed with PBS, and further incubated with 0.25 mL of complete medium for 24 h. Finally, cells were washed with PBS and lysed with Glo-lysis buffer (10 min, 25 °C). The luciferase activity was measured after cell lysate centrifugation (4,500 rpm, 2 min) using the ONE Glo luciferase assay following manufacturer’s instructions. The relative luminescence units (RLU) were measured using a Synergy2 Biotek plate reader (Gen5 data acquisition software), and normalized against the total protein content (BCA assay) for each well.

### Statistical analysis

Stability and silencing data were analysed using a two-sample *t*-test using the Welch correction (i.e., without assuming equal variance). If *p* > 0.05, no statistical difference was assumed; in the figures * means *p* < 0.05, ** means *p* < 0.01, *** means *p* < 0.001, **** means *p* < 0.0001.

## Results and Discussion

### Template vs direct complexation: physico-chemical comparison

#### Similarities

**Figure 1:**
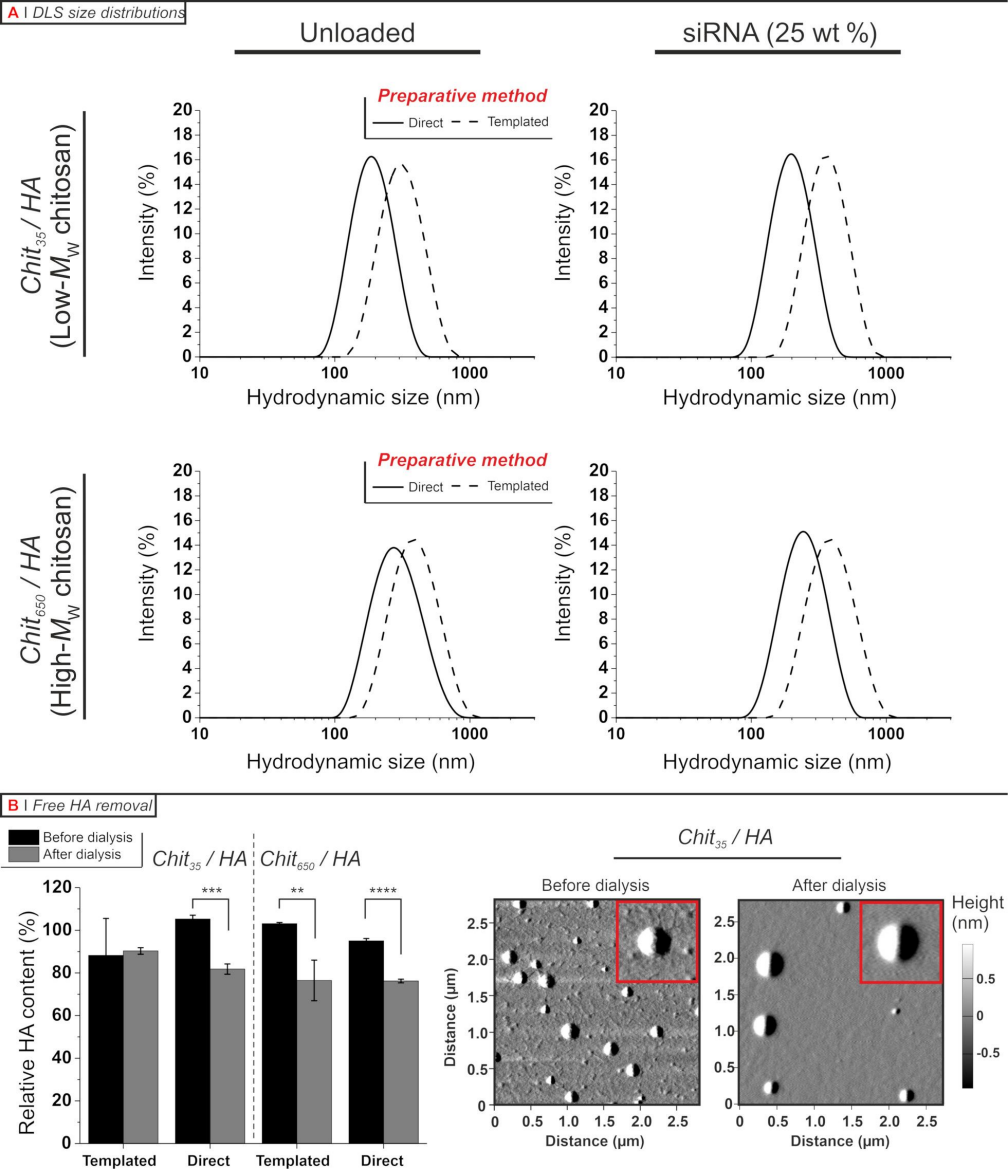
A) Size distribution of chitosan/HA nanoparticles (1 mg/mL, deionized water) prepared from 35 (top) and 650 (bottom) kDa chitosan using a templated (dashed lines) or direct (solid lines) complexation in the absence (left) or presence (right) of siRNA (25 wt % in relation to chitosan). In this and later figures Chit_35_ and Chit_650_ are, respectively, referred to as low-*M*_W_ and high-*M*_W_ chitosan to avoid the use of subscripts for a better readability. B) Quantification of free HA after nanoparticle preparation. Left: The HA content of the different nanoparticle formulations (1 mg/mL, deionized water) was measured by capillary electrophoresis before and after dialysis (MWCO = 1,000 kDa). Results are expressed as the relative amount of complexed HA (incorporated in the nanoparticle) relative to the total amount of HA in the nanoparticle feed (*n* = 3). Right*:* AFM amplitude images of (low-*M*_W_ chitosan) nanoparticles after dialysis showing the complete removal of unbound HA (absence of “debris” material on the mica surface).

The two preparative methods yielded broadly similar nanoparticles (Table 1). Firstly, no significant difference can be seen in their ζ-potential values (strongly negative). Chit_650_ yielded particles marginally larger than Chit_35_, and the width of the particle size distribution – as assessed with DLS as a stand-alone instrument – was also similar (Figure 1, compare dashed and solid lines). Further, both methods allowed for quantitative siRNA entrapment, and the encapsulation did not significantly affect the nanoparticle size (up to a loading of 25 wt % in relation to chitosan; Figure 1 and Table 1). Last, also the stability of the nanoparticles was similar: the behaviour of the nanoparticles prepared by the direct method upon dialysis, storage and dispersion in different media (Supporting Information File 1, Section SI2 and Figure S1) was comparable to that previously reported by our group for the templated method [18].

**Table 1:** Physico-chemical characterization of the nanoparticles^a^.

	Z-average size (nm)	PDI^b^	ζ-potential (mV)	siRNA loading		C/N^e^	P/N^e^
				Size incr.^c^	EE^d^		

Chit_35_-TPP	166 ± 5	0.17 ± 0.05	+37 ± 1		>99%	6.16 (5.72)	0.48 (0.36)
Chit_35_/HA_templ_	310 ± 50	0.17 ± 0.06	−38 ± 5	3%	>99%	11.56 (9.71)	nd (0.11)
Chit_35_/HA_dir_	220 ± 30	0.19 ± 0.07	−39 ± 2	0%	>99%	10.08 (9.28)	nd (0)

Chit_650_-TPP	368 ± 15	0.28 ± 0.01	+50 ± 2		>99%	6.49 (5.72)	0.31 (0.36)
Chit_650_/HA_templ_	320 ± 30	0.17 ± 0.06	−38 ± 4	6%	>99%	11.91 (9.71)	nd (0.11)
Chit_650_/HA_dir_	260 ± 40	0.20 ± 0.05	−40 ± 2	0%	>99%	9.7 (9.28)	nd (0)

^a^In deionized water, room temperature, concentration of 1 mg/mL. Data are averages ± standard deviation from three separate preparations. ^b^PDI: polydispersity index. ^c^% of increase in the nanoparticle Z-average size with a 25 wt % (in comparison to chitosan) siRNA loading. The ζ-potential did not appreciably vary upon siRNA loading (−39 to −41 mV for all chitosan/HA nanoparticles). ^d^EE: the encapsulation efficiency of siRNA (expressed in wt % in reference to siRNA feed) refers to a loading of 25 wt % in relation to the amount of chitosan. ^e^In brackets the theoretical values, which are obtained assuming that all components are quantitatively entrapped in the particles. Please note that our detection limit for P/N is around 0.07.

Very interestingly, the two preparative methods provided particles with a very similar composition. Here, we have normalized the carbon and phosphorous content to that of nitrogen (last two columns in Table 1). TPP has no carbon or nitrogen, therefore the C/N ratio is a reporter of the chitosan/HA composition (see, e.g., how different this indicator is for chitosan alone and for chitosan/HA), and the P/N ratio is a reporter of the TPP content. Please note that the presence of water in the samples does not affect either of these indicators. Independent of the preparative method, the final particles have the same chitosan/HA ratio (the C/N ratio is indistinguishable) and are devoid of TPP (non-detectable phosphorous, differently from chitosan/TPP particles). The latter point indicates a quantitative displacement of TPP by HA, the driving force of which is the higher avidity of the polysaccharide. For example, the individual phosphate groups of siRNA have most likely the same affinity for chitosan as those of TPP, but siRNA has surely a larger avidity due to its polymeric structure and therefore it is not displaced by HA. We cannot, however, exclude the siRNA/chitosan interactions to be somehow weakened by the presence of HA. The TPP–HA exchange may also be facilitated by the rather low degree of protonation of chitosan, which make its electrostatic interactions more easily reversible.

Finally, the two processes were similar in terms of the presence of unbound HA. CD44, the HA main biological target, is a saturable receptor [28–29], and the initial binding of HA species with molecular weights greater than 30 kDa to CD44 is described as essentially irreversible [29]. It is therefore important to assess whether in the same formulation HA nanoparticles are present together with unbound HA, which could potentially reduce binding and efficacy of the payload-carrying nanoparticles. Using AFM, we have shown that dialysis through membranes with a large MWCO (1,000 kDa) completely removes soluble HA (Figure 1B, right). When analysing the particles before and after dialysis, we saw no significant difference in HA nanoparticle incorporation (always at least 80 wt % of the feed) between the two chitosan polymers of different MW or the two preparative methods (Figure 1B, left).

#### Differences

The most important difference between the two methods is that the template process yielded larger nanoparticles, either with or without siRNA. We are inclined to ascribe the larger size to aggregation occurring during the TPP–HA exchange, since the ζ-potential inversion (from the positively charged chitosan/TPP to the negatively charged chitosan/HA nanoparticles) implies intermediate stages with negligible electrostatic repulsion.

**Figure 2:**
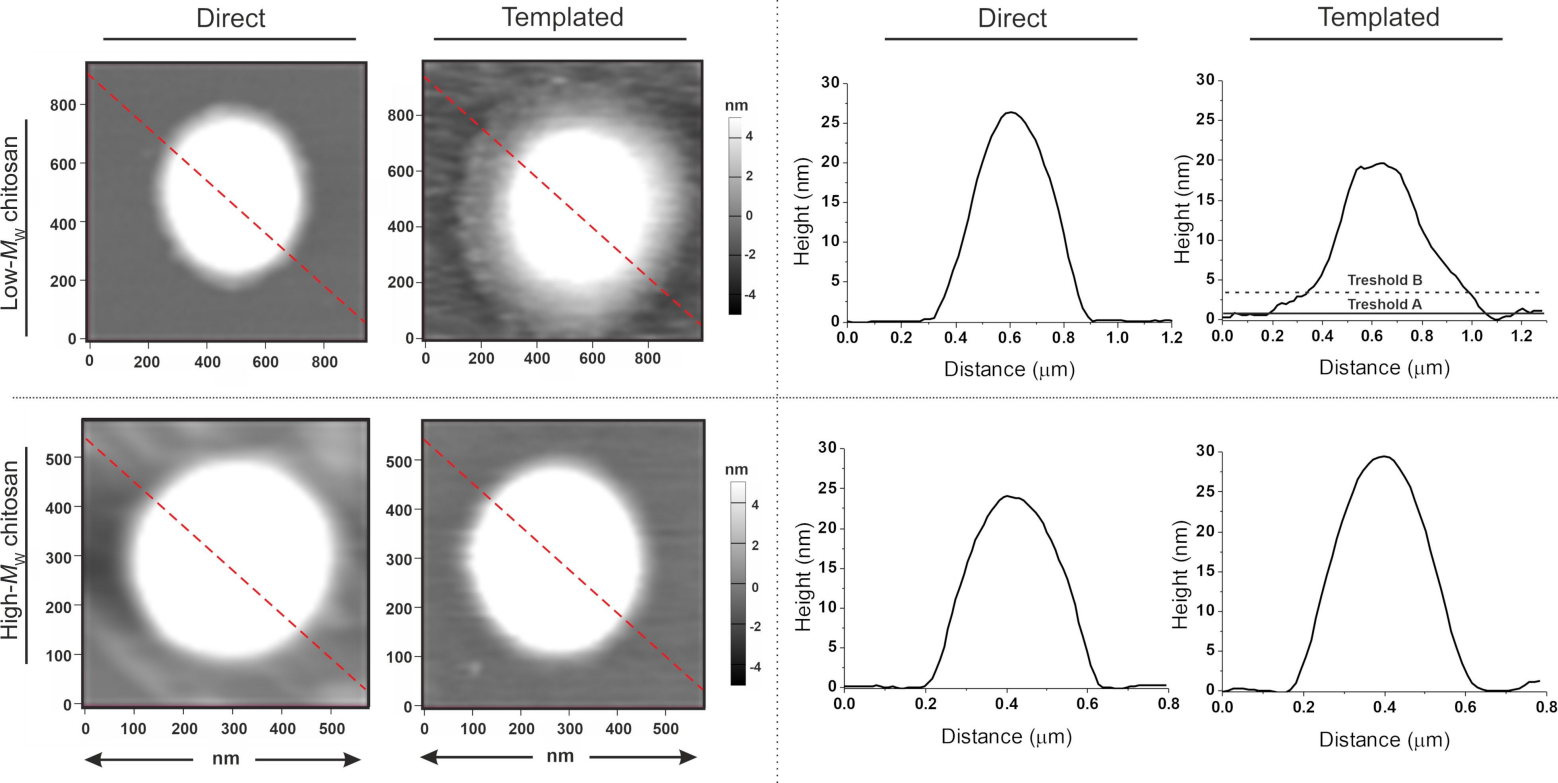
Tapping-mode AFM height images of chitosan/HA dried on mica (left) and height profiles corresponding to the red dashed lines in the images (right). For the templated Chit_35_/HA nanoparticles two thresholds were used to calculate separately the volume distributions of the nanoparticle corona and core. Please note that the HA corona is even more visible in amplitude images, see Supporting Information File 1, Figure S2.

We have previously shown that in the template process, and only with low-MW chitosan, the particles show a sort of HA corona [18–19]; this does not occur in the direct complexation (Figure 2).

In our original interpretation, we ascribed the HA corona formation to a difficult penetration of HA into the Chit_35_/TPP matrix, which is more densely cross-linked than Chit_650_/TPP (lower entropic penalty to pay for un-coiling lower-MW macromolecules). In the light of the TPP–HA exchange, this interpretation must be revised: the same factor (better packing of Chit_35_) increases the chitosan concentration in the bulk of the templating particles, potentially leading to a tighter HA complexation in the bulk, but also to a reduced surface charge and to a lighter surface complexation. This should also cause a larger extent of aggregation. Conversely, in the absence of a dense chitosan pre-packing (high-MW chitosan, or direct complexation), no corona would form but the particles would be expected to be less aggregated and more compact. In order to shed further light on the nanoparticle morphology we have employed field flow fractionation, using both static (AF4-SLS) and dynamic (AF4-DLS) light scattering detectors. In addition to more accurate size distributions due to the fractionation prior to the in-line analysis, this combination provides also information about the colloid compactness through parameters such as their average radius of gyration *R*_g_ (it depends on the mass distribution), the shape factor ρ defined as the *R*_g_/*R*_H_ ratio, and the mass fractal dimension *D*_m_ defined as the exponent in the relation between mass of a colloidal object *M* and its *R*_g_, 
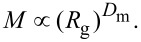
 The last two parameters are particularly important: A) ρ = 0.77 for a perfect sphere, ρ = 1.15–1.2 for random coils under θ-conditions and increasing with increasing solvation [30], i.e., it typically increases with decreasing compaction and statistical fluctuations. B) *D*_m_ expresses the scaling between the mass of a colloid and its dimension, therefore is a direct measure of its compactness; for example, for a “dense solid” colloid, its value will approach 3, while its values decrease for aggregates, and therefore this parameter has been used for aggregation studies of, e.g., liposomes [26], amphiphilic polymers [31], and gold nanoparticles [32]. We refer the reader to the excellent review of Bushell in 2002 for an extensive physico-mathematical explanation of *D*_m_ [25].

**Figure 3:**
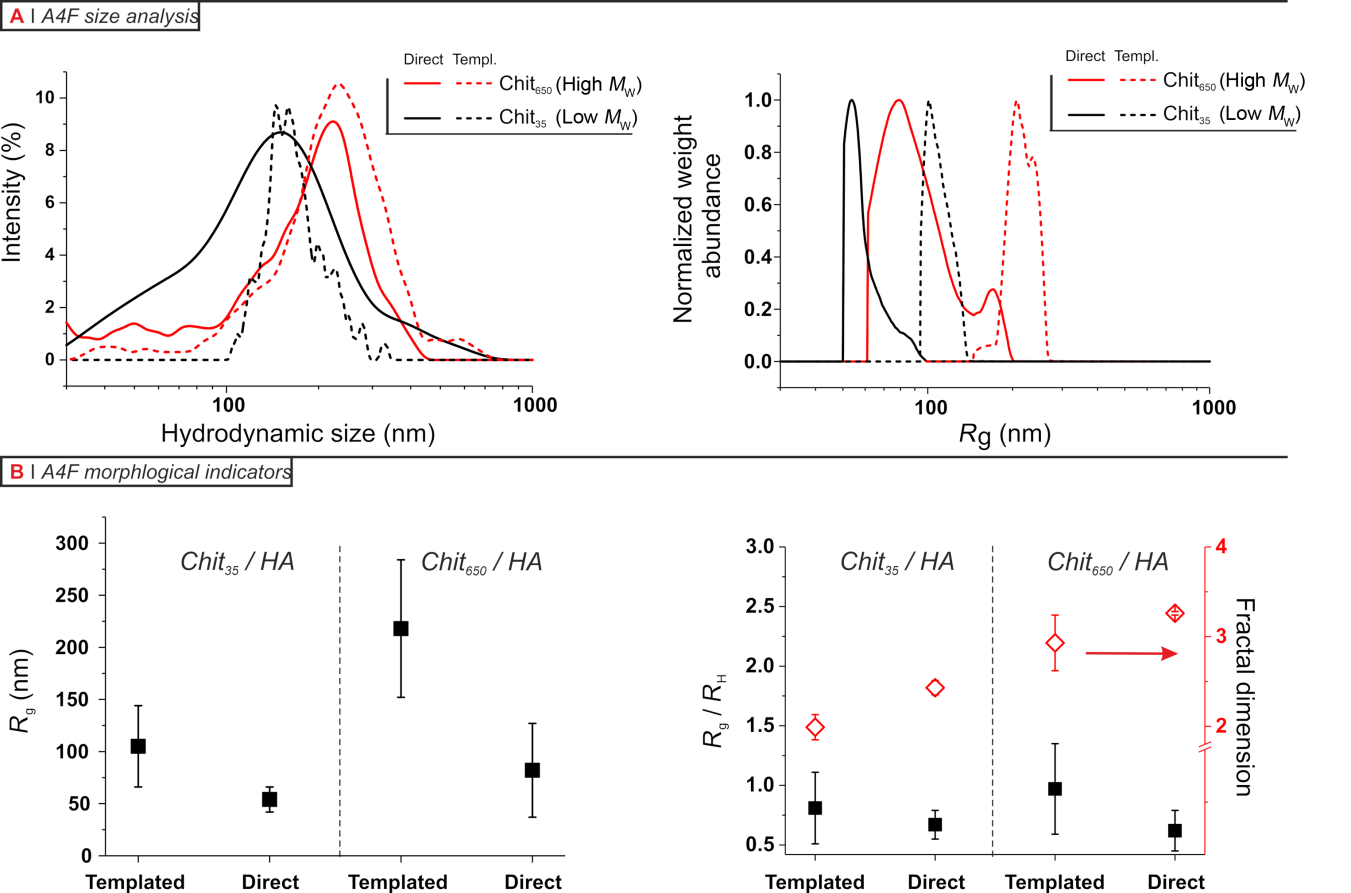
A) Asymmetric-flow field flow fractionation (AF4) characterization of nanoparticles (see also Supporting Information File 1, Figure S3 for examples of AF4 elugrams). Left: hydrodynamic size distributions from in-line DLS. Right: *R*_g_ distributions from in-line SLS. B) Morphological indicators obtained from AF4 analysis. Left: Weight-average *R*_g_ values and Right: mass fractal dimension and *R*_g_/*R*_h_ ratio for the four kinds of chitosan/HA nanoparticles. Please note that the error bars in the first graph are the widths at half maximum of the log-normal fit of the differential *R*_g_ distributions, in the second graph are the errors of the fitting.

Firstly, the AF4-DLS curves (Figure 3A, left) largely overlapped. Thus, it appears that DLS alone (Figure 1) overestimated the difference in hydrodynamic size between the two preparative methods (and overestimated the size itself, possibly due to the disproportionate weight of the scattering from large nanoparticles). Yet, small differences in this sense can still be seen and indeed this effect is confirmed by the distributions obtained through AF4-SLS (Figure 3A, right).

Secondly, there is a clear indication for a less compact structure of the templated particles, which have larger *R*_g_ values (Figure 3B, left) but a hydrodynamic size still relatively comparable to that of the particles obtained through direct complexation. This is summed up by the aspect ratio values (black squares in Figure 3B, right): ρ ranges from 0.6–0.8 for nanoparticles from direct complexation (essentially compact spheres), to 0.8–1 for the products of the template process; the latter are compatible with particles having a spherical shape but a relatively looser internal structure.

An even clearer result is provided by the *D*_m_ values (red diamonds in Figure 3B, right). *D*_m_ is always ≥2, indicating a spherical shape (in agreement with ρ values), but is considerably smaller for the template process. Interestingly, chitosan/HA and chitosan/TPP nanoparticles from direct complexation have almost identical *D*_m_ values (both around 3.1–3.2 for Chit_650_ and 2.4–2.5 for Chit_35_), indicating a similarly compact nature. On the contrary, the template chitosan/HA particles had significantly lower *D*_m_ values, in particular Chit_35_. This means that the particles displaying an HA corona are also the least compact, which corroborates the above hypothesis for the corona formation (small compact particles connected by loosely bound HA).

### Evaluation of CD44-targeted delivery of siRNA

**Figure 4:**
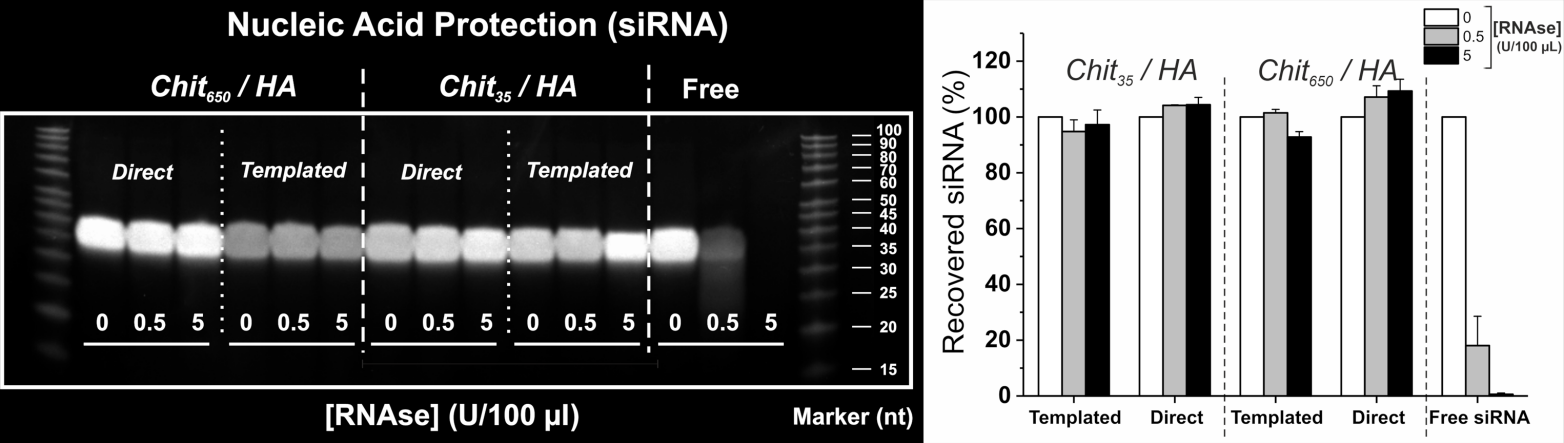
Protection of siRNA payload from enzymatic degradation. Left: PAGE analysis of entrapped siRNA after nanoparticle incubation with increasing concentrations of RNAse I, followed by quenching of nucleases, chitosanase and heparin treatment. Free, non-encapsulated siRNA was used as a control. The siRNA content in the particles was 25 wt % in relation to chitosan. Right: Plot of the band intensities relative to the signal of the negative control (no RNAse I treatment) for each formulation. Error bars represent standard deviation (*n* = 3). Please note that results are normalized against the amount of siRNA released from formulations incubated with no RNase I (non-degraded) to account for any dilution factor or lose of material, e.g., note the fainter bands detected for templated high-*M*_W_ chitosan nanoparticles.

One of the most fundamental aspects to a successful intracellular siRNA delivery is the ability of the carrier to protect the cargo from enzymatic degradation to allow its release in the cytoplasm, where the RNA machinery is located. Hence, as a first step we ruled out any differences in the protective behaviour of our nanoparticles between preparative methods (Figure 4). Specifically, siRNA-loaded nanoparticles were incubated in the presence of RNAse I at concentrations sufficiently high to degrade partially (at 0.5 U) or completely (at 5 U) the same amount of non-encapsulated nucleic acid (labelled as “free” in Figure 4). RNA was then decomplexed by enzymatically digesting chitosan and further displacing its fragments with heparin (more strongly anionic than HA and RNA), and finally analysed by gel electrophoresis. The central point is that all nanoparticles protected their payload from RNAse.

Secondly, we evaluated the biocompatibility of these nanoparticles using two cellular models. These models were murine RAW 264.7 macrophages (already used in our previous studies with chitosan-TPP/HA nanoparticles [19–20]) due to their relatively high CD44 expression [33], and the human colorectal HCT-116, a CD44-overexpressing colorectal line the suitability of which for HA-based targeting therapies has already been reported [22,34–35]. It is worth mentioning that HCT-116 apparently shows a lower CD44 expression (see Supporting Information File 1, Section SI5), but this does not imply a lower CD44 endocytic activity.

**Figure 5:**
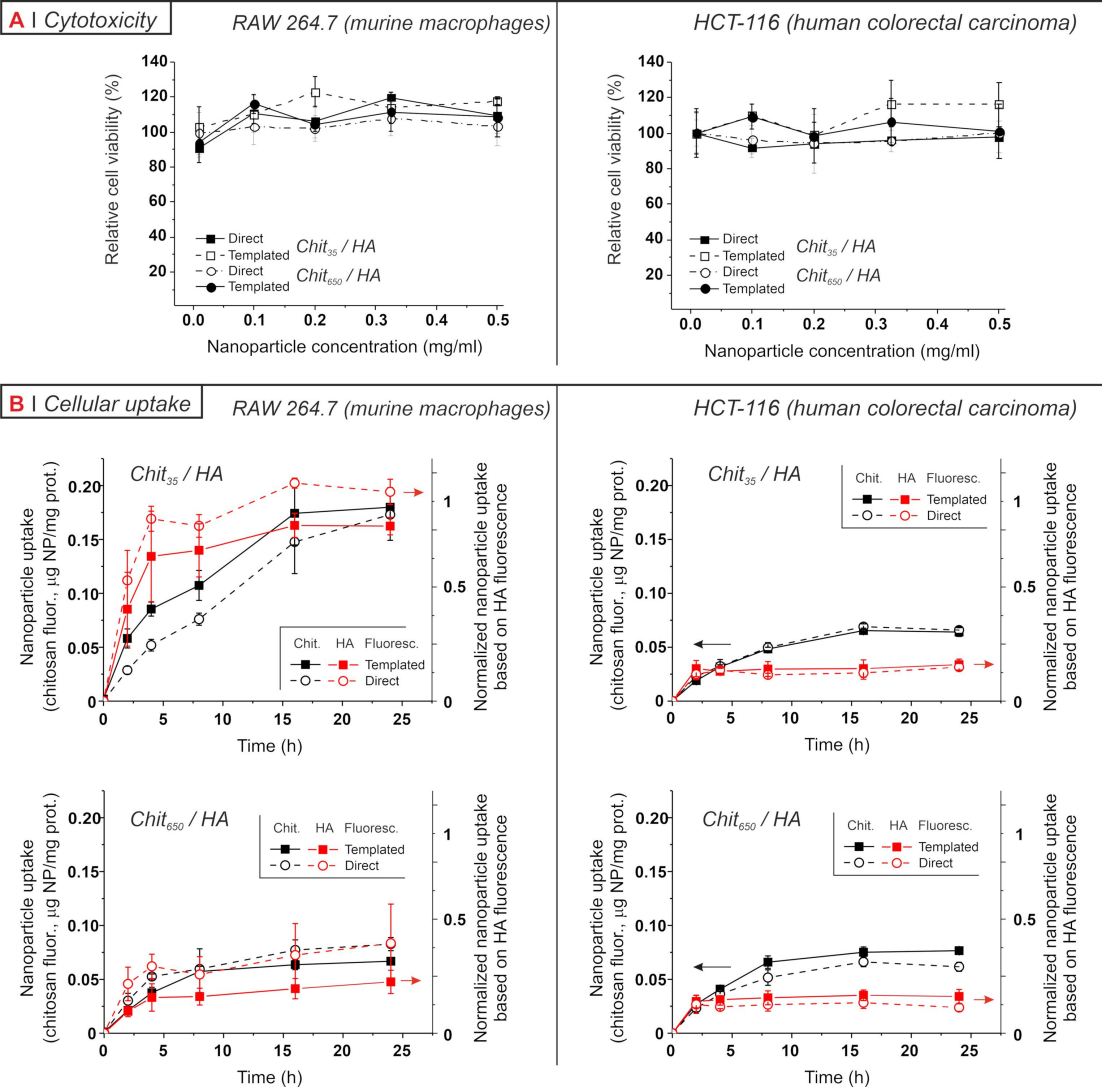
A) Relative cell viability of RAW 264.7 (left) and HCT-116 (right) cell lines as a function of nanoparticle concentrations (0.01–0.5 mg/mL, 24 h incubation). Percentages are relative to the normalized mitochondrial activity of untreated cells. The differences between preparative methods are not statistically significant. B) The cellular uptake of nanoparticles was followed by measuring the fluorescence of lysates after cells were incubated with particles containing RITC-labelled chitosan or rhodamine-labelled HA. The lysate fluorescence is a quantitative measure of the nanoparticle uptake (bound and internalized particles), and the data are first calibrated with nanoparticles dispersed in cell lysates to obtain quantities of nanoparticles and then normalized against the cell protein content to finally obtain values of the amount of nanoparticles uptaken “per cell”. It is noteworthy that for any given data point the uptake measured from HA-associated fluorescence was consistently lower than that obtained via chitosan fluorescence. Since the fluorophores are essentially the same, this may be due to a detachment of the two components upon uptake, because the quantum yield of the fluorophores in isolated chitosan or HA and in nanoparticles may be significantly different. For comparison, we have therefore further normalized the data from HA fluorescence to the average value of Chit_35_/HA nanoparticles in RAW macrophages after 24 h. In all graphs, error bars represent standard deviation (*n* = 3).

The cytotoxicity of the nanoparticles was assessed using the MTS assay, a colourimetric method that measures mitochondrial metabolic activity (data normalized against the protein content, assumed roughly proportional to the cell number). Independently of the preparative method and the MW of chitosan, all formulations had a negligible effect on the cell viability up to 0.5 mg/mL in both models (Figure 5A). Despite the fragile nature of macrophages, the low toxicity seen for HA-coated chitosan nanoparticles is in accordance with what reported in RAW 264.7 macrophages for other HA-based nanomaterials, such as HA-coated liposomes [36] and a library of lipid nanoparticles with surface-anchored HA [37], or chitosan-based carriers, such as mannosylated chitosan nanoparticles [38] or siRNA-entrapped chitosan nanoparticles (with or without TPP) [39]. The innocuous character of HA-coated chitosan nanoparticles in HCT-116 is also consistent with previous studies on HA-based cationic nanocarriers [40–41].

Next, we analysed the nanoparticle uptake in the two cell lines for up to 24 h; we tracked the fluorescence associated to nanoparticles in cell lysates, which accounts for both membrane-bound and internalized materials [10]. We used fluorescently labelled chitosan and HA, producing nanoparticles selectively containing one labelled polymer. Following either chitosan- or HA-associated fluorescence (respectively black and red symbols in Figure 5), we observed qualitatively similar uptake kinetics, which is a sign that the two components are mostly internalized together and therefore indicates a reasonable stability of the particles until binding to cells. However, it is also clear that nature and extent of particle–cell interactions are cell type-dependent, see for example the different kinetics of Chit_650_/HA in macrophages and in HCT-116.

It is immediately apparent (Figure 5B) that the uptake kinetics was not influenced by the preparative method. With both cell lines and both chitosan MW, the particle uptake kinetics showed evidence of saturation and was not affected by the preparative method. We have previously reported that RAW 264.7 macrophages take up differently nanoparticles prepared from chitosan with low and high molecular weight, with the former reaching considerably lower saturation levels than the latter, although more rapidly [14]. We then ascribed this effect to a higher avidity of HA for its receptors provided by the corona arrangement, summarized as “stronger interactions – more receptors clustered around each particle – fewer particles internalized”. Here, we confirmed this behaviour in macrophages. However, A) it was recorded also for directly complexed particles (= it is a general feature of Chit_35_/HA complexes), and B) the uptake of all particles in HCT-116 was essentially identical. These two observations seem to discount both the hypothesis of the HA corona as a controlling factor and the possibility that particle compactness plays any major role.

In order to have a more complete overview of the nanoparticle behaviour, we have evaluated their silencing efficiency when loaded with a functional anti-luciferase (anti-Luc) siRNA. To this end, cells were first pre-transfected with a luciferase-encoding plasmid (pGL3) using low-toxicity Lipofectamine (LTX) as a vector, and then treated with anti-Luc siRNA vectored either in nanoparticles or LTX complexes in order to silence luciferase expression. For comparison purposes, we reproduced the experimental conditions previously reported by our group for templated chitosan/HA particles (i.e., delivery of 200 nM anti-Luc per well) on RAW 264.7 macrophages, which are difficult to transfect [15].

**Figure 6:**
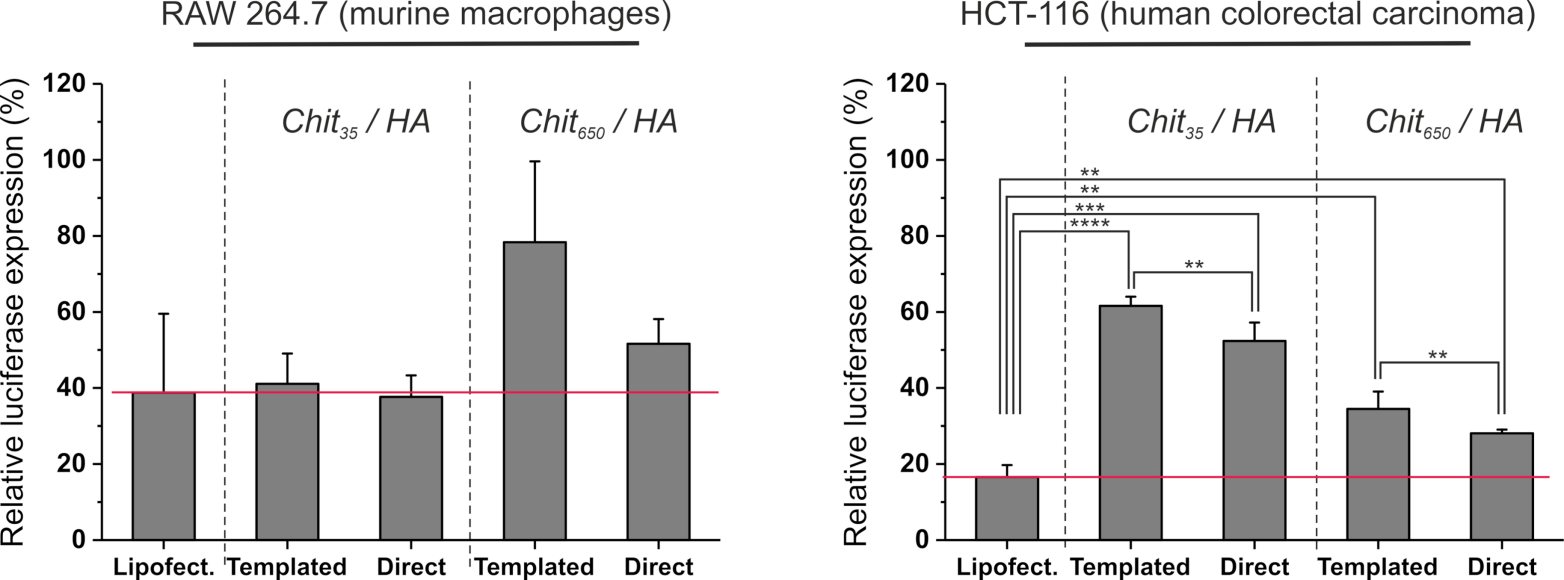
siRNA-mediated luciferase silencing using nanoparticles obtained via a templated or direct complexation method. The results are expressed as the percentage of luciferase expression relative to the average RLUs of the negative control (i.e., cells pre-transfected with the pGL3 plasmid DNA, without anti-Luc siRNA treatment) (*n* = 3). Statistical analysis (T-test, two-tailed) showed no significant differences between preparative methods in RAW macrophages, but a somehow better silencing efficiency of direct complexation (and of high-*M*_W_ chitosan) in HCT-116.

Under these conditions, we observed a distinct cell-specific behaviour: A) in macrophages (Figure 6, left), templated Chit_35_/HA particles were internalized less but silenced more than templated Chit_650_/HA, as previously reported [14–15]. The directly complexed Chit_35_/HA particles were again internalized less than Chit_650_/HA, but the increase in silencing was marginal. B) In HCT-116 (Figure 6, right), the preparative method played little role. Very differently from macrophages, Chit_35_/HA particles were internalized similarly Chit_650_/HA, but silenced less.

Besides the obvious conclusion that Chit_650_/HA particles are suitable for HCT-116 and Chit_35_/HA particles are suitable for macrophages, the overall behaviour seems rather erratic, but there are directions for a logical explanation.

First, the two cell lines surely differ in CD44 expression, clustering and endocytic role. For example, HCT-116 cells massively express CD44, both in its standard and variant forms [42]. Their “HA receptor cocktail” may trigger internalization processes with conditions that favour the endosomolytic properties of high-MW chitosan. High-MW polycations should, in principle, be better at membrane disruption. RAW macrophages, on the contrary, may bind and internalize HA through a different “receptor cocktail”. For example, these macrophages are commonly used as a toll-like receptor 4 (TLR4)-positive model in inflammation, and HCT-116 as a model for limited TLR4 signalling [43]. Further, TLR4 is involved in the modulation of lipid raft-associated interactions between HA and CD44 [44–45]. Therefore, a receptor such as TLR4 might be involved in HA internalization in RAW macrophages and not in HCT-116. In RAW 264.7 macrophages CD44 has been described not only as an endocytic receptor, but also as a fully competent phagocytic receptor for the digestion of large particles [46].

It is therefore not unlikely that the macrophagic “HA receptor cocktail” triggers intracellular processes different from those in HCT-116; for example, in macrophages siRNA liberation from its complex with chitosan may be the controlling step, instead of endolysosome disruption. Under this hypothesis, Chit_35_ has a lower avidity for siRNA [4], and this may be the reason for its better performance. Following this hypothetical train of thoughts, the very low silencing efficiency of templated Chit_650_/HA would be ascribed to the template process yielding aggregates of smaller and possibly more compact particles that may be particularly resistant to siRNA liberation. Indeed, templated particles in general showed a poorer silencing performance, albeit in some cases only slightly. In short, a different controlling factor (e.g., membrane disruption for HCT-116, siRNA release for RAW macrophages) may explain most of the differences seen in the silencing activities.

## Conclusion

We have evaluated the effects of the preparative method of nanoparticles (template vs direct, with two chitosan polymers of different MW) on the morphology of the nanoparticles and on their in vitro performance as carriers.

Although both processes are characterized by a simple add–mix procedure of aqueous solutions, the absence of purification (dialysis) steps in the direct complexation method is particularly appealing. Besides the shorter time of preparation, this allows for an easier implementation of aseptic manufacturing conditions.

Moreover, on one hand, the two production processes provided broadly similar nanoparticles with sizes always well below 500 nm, HA on their surface, negligible toxicity, and effective encapsulation and protection of RNA. On the other hand, these particles differed in their dimensions and, above all, in their internal compactness. The template process implied significant aggregation, which we ascribed to the step of TPP–HA exchange. Confirming our previous observations, templated particles based on low-MW chitosan showed an HA corona, which was not present in the directly complexed particles with the same composition.

The in vitro performance was assessed by monitoring uptake and silencing efficiency of both nanoparticle components (HA and chitosan) in two cell lines, leading to the following observations: A) a better silencing performance for low-MW chitosan in RAW macrophages, and for high-MW chitosan in HCT-116; B) a higher uptake of high-MW chitosan-based particles in RAW macrophages, which – together with the poor silencing efficacy – may suggest the involvement of a different internalization machinery; C) the irrelevance of the HA corona for the nanoparticle uptake, and the slightly poorer performance of templated particles in silencing. However, one should extrapolate the irrelevance of HA corona for cell–nanoparticle interactions: in a previous study we have demonstrated that it does allow for a better exposure of HA-bound ligands [16].

The more general conclusion, however, is the strongly cell-dependent nature of the effects that nanoparticle morphology may have on nanoparticle internalization and silencing performance (see point A in the previous paragraph). This highlights the need of a better understanding of the cell-specific binding and trafficking event for a prediction of the therapeutic efficacy of a nanocarrier.

## Supporting Information

File 1 Additional experimental description and data.
