# Supplementary material for: The different ways to chitosan/hyaluronic acid nanoparticles: templated vs direct complexation. Influence of particle preparation on morphology, cell uptake and silencing efficiency
Source: Beilstein J Nanotechnol. 2019 Dec 30;10:2594–608. doi: 10.3762/bjnano.10.250 (PMC6964650; doi:10.3762/bjnano.10.250)
Supplement: File 1 — Additional experimental description and data. [file Beilstein_J_Nanotechnol-10-2594-s001.pdf]

BEILSTEIN JOURNAL OF NANOTECHNOLOGY

Supporting Information

for

The different ways to chitosan/hyaluronic acid nanoparticles: templated vs direct complexation. Influence of particle preparation on morphology, cell uptake and silencing efficiency

Arianna Gennari, Julio M. Rios de la Rosa, Erwin Hohn, Maria Pelliccia, Enrique Lallana, Roberto Donno, Annalisa Tirella and Nicola Tirelli

Beilstein J. Nanotechnol. **2019,** *10,* 2594–2608. doi:10.3762/bjnano.10.250

Additional experimental description and data

License and Terms: This is a supporting information file under the terms of the Creative Commons Attribution License (https://creativecommons.org/licenses/by/4.0). Please note that the reuse, redistribution and reproduction in particular requires that the authors and source are credited.
The license is subject to the *Beilstein Journal of Nanotechnology* terms and conditions: (https://www.beilstein-journals.org/bjnano)

SI1 Additional experimental descriptions

SI1.1 Materials

SI1.1.1. Chemicals

1 M hydrochloric acid (HCl, J/4320/15), 1 M sodium hydroxide (NaOH, J/7620/15), dimethyl sulfoxide (DMSO, D/14120/PB08) and 1,2-propanol (P/7490/17) were all purchased from Thermo Fisher Scientific (Loughborough, UK). Sodium triphosphate pentabasic (TPP, #72061), rhodamine B isothiocyanate (RITC, #283924), 4-(2-hydroxyethyl)-1-piperazineethanesulfonic acid (HEPES, #90909C), BCA assay kit (BCA1 and B963), phosphate buffered saline (PBS, P4417), sodium azide (S2002) and RIPA buffer (R0278) were obtained from Sigma-Aldrich (Gillingham, UK). Glacial acetic acid and sodium acetate were purchased from VWR BDH Chemicals (Poole, UK). Chitosanase from *Streptomyces griseus* and RNAse I were purchased from Merck (Nottingham, UK). RNA Low Molecular Weight Marker Ladder (10-100 nt), GelRed™ nucleic acid gel stain, and Quant-iT™ RiboGreen® RNA assay kit reagent were purchased respectively from Affymetrix (High Wycombe, UK), Biotium (CA, USA) and Molecular Probes (OR, USA). UltraPure™ DNase/RNase-free distilled water and low toxicity Lipofectamine® LTX reagent were purchased from Invitrogen (Paisley, UK). CellTiter 96® AQueous One Solution Cell Proliferation Assay – MTS (3-(4,5-dimethylthiazol-2-yl)-5-(3-carboxymethoxyphenyl)-2-(4-sulfophenyl)-2*H*-tetrazolium), pGL3 - Control Luciferase Reporter Vector, and ONE Glo Luciferase Assay System were purchased from Promega (WN, USA). Anti-Luc siRNA-1 (D-002050-01-05) with target sequence 5'-GAT TAT GTC CGG TTA TGT ATT-3' was purchased from GE-Dharmacon (Epsom, UK).

SI1.1.2. Cell Culture

The human colorectal cancer cell line HCT-116 (CCL-247™) and the murine macrophagic cell line RAW 264.7 (TIB-71™) were purchased from ATCC (Maryland, USA) and cultured respectively in McCoy's 5A (M8403) or high glucose DMEM (D5671), each supplemented with 10% (v/v) foetal bovine serum (FBS) (F7524), 2 mM L-Glutamine (G7513), and 1% (v/v) Penicilin-Streptomycin (P4333). DMEM, high glucose powder (D5648) and McCoy's 5A powder (M4892) were also used to prepare double concentrated media. All products were purchased from Sigma-Aldrich (Gillingham, UK). Disposable Sterile Filter Systems (0.22 µm), cell culture flasks and well plates were purchased from Corning (Deeside, UK).

SI1.2 Procedures for fluorescent labelling

SI1.1.1. Chitosan labelling with rhodamine isothiocyanate (RITC)

100 mg of $Chit_{35}$ or $Chit_{650}$, corresponding to 0.5 mmol of glucosamine (Glu) units, were dissolved in 18 ml 0.1 M acetic acid (aq) overnight and the pH was then adjusted to 4 with 0.1 M NaOH (aq). 8 mg of RITC (0.015 mmol of isothiocyanate groups) dissolved in 1.6 ml of dry DMSO were then added dropwise to the above chitosan solution. The reaction mixture was stirred (300 rpm) overnight at 25 °C in the dark. Afterward, the reaction solution was diluted with 19.6 ml of deionized water and purified via centrifugal ultrafiltration against deionized water using Amicon Ultra-4 devices (MWCO 50 kDa for high MW chitosan, 10 KDa for low MW chitosan) until the conductivity and pH values of the wastewater reached those of deionized water. Finally, the chitosan-RITC samples were freeze-dried and stored at 4 °C (Mass recovery = 85%). The degree of functionalization was determined by measuring the fluorescence intensity of the RITC-labeled chitosan products; using a calibration curve of free RITC to link the emission to the molar concentrations of the fluorophore per gram of functionalized polymer and then further convert it in a molar ratio between functionalized and total number of Glu units in the chitosan. Typical degree of derivatization achieved is 0.25-0.30% mol of Glu units.

SI1.1.2. Hyaluronic acid labelling with rhodamine

Hyaluronic acid (HA; $\overline{M_w}$ = 180 kDa, assessed by GPC) was provided by Novozymes (Bagsvaerd, Denmark) and covalently conjugated to Lissamine™ Rhodamine B Ethylenediamine (Thermo Scientific, UK). All solutions were prepared in 100 mM HEPES buffer at pH 7.4 unless stated otherwise. In a similar manner as described in [1], 150 mg of HA (0.5 mmol of carboxylate) were dissolved in 15 ml by shaking overnight. After complete dissolution of HA, 3.75 ml of a solution containing 4.32 mg of Rhodamine were added followed by 3.75 ml of a 65 mM 4-(4,6-dimethoxy-1,3,5-triazin-2-yl)-4-methylmorpholinium chloride (DMT-MM) solution (Sigma-Aldrich, UK). The reaction was stirred (at 300 rpm) for 24 h at 25 °C and then quenched and precipitated using a 20-fold volume excess of ethanol (96% v/v). The mixture was further incubated overnight at 4 °C to ensure the complete precipitation of the labelled HA. The precipitate was collected after centrifugation (10 min at 4500*g*), dissolved in 15 ml of deionized water, and purified by dialysis (MW cut-off 20 kDa). Finally, the HA solution was freeze-dried. Mass recovery: 74%. Degree of derivatization: 0.7% mol (calculated by measuring the fluorescence of Rhodamine-conjugated HA (Ex: 540/25, Em: 620/40 nm) and comparing it to a calibration with free Rhodamine).

SI2. Physico-chemical characterization

Table S1: Physico-chemical characteristics of nanoparticles in deionized water, room temperature, concentration of 1 mg/ml. Data are reported as averages ± standard deviation from three independent preparations (n = 3 each).

	**- siRNA**		**+ siRNA (25% wt.)**	
Chitosan MW	35 kDa	670 kDa	35 kDa	670 kDa
Template method				
Z-average size (nm)	310 ± 50	320 ± 30	320 ± 70	340 ± 60
PDI[a]	0.17 ± 0.06	0.22 ± 0.06	0.20 ± 0.04	0.24 ± 0.05
ζ-potential (mV)	-38 ± 5	-38 ± 4	-41 ± 5	-39 ± 6
EE[b] (% wt.)	-	-	>99	>99
Direct complexation				
Z-average size (nm)	220 ± 30	260 ± 40	220 ± 20	260 ± 30
PDI	0.19 ± 0.07	0.20 ± 0.05	0.21 ± 0.05	0.23 ± 0.04
ζ-potential (mV)	-39 ± 2	-40 ± 2	-40 ± 2	-40 ± 2
EE[b] (% wt.)	-	-	>99	>99

[a] PDI = polydispersity index; [b] EE= encapsulation efficiency.

SI2.1 Nanoparticle Stability

The stability of high and low MW chitosan/HA nanoparticles was checked against:
A and B) Dialysis and storage: after preparation the particles were dialyzed against MilliQ water for 5 hours changing the water every 20 minutes. Aliquots of both dialyzed and non-dialyzed particles were then stored at 4 °C for 14 days and their size and surface charge was checked by DLS as a measure of nanoparticle stability over time.

C) Dilution with different media: 1 ml of freshly prepared nanoparticles was diluted with an equal volume of either MilliQ water, 2X saline (i.e. 1.8% w/v NaCl), 200 mM acetate buffer at pH 5 or 2X PBS, their hydrodynamic size and zeta potential were then measured by DLS.

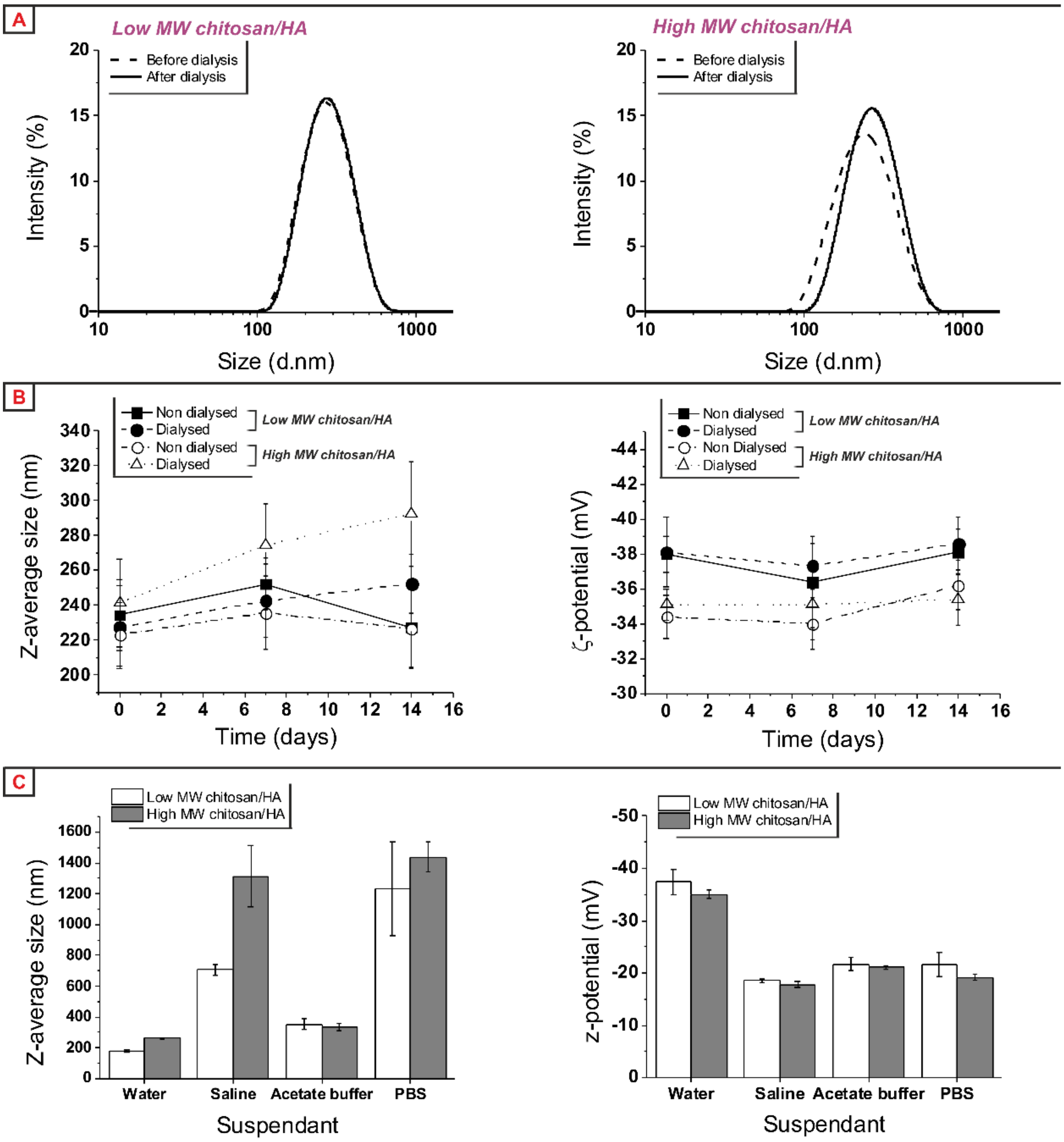

Figure S1: Dimensional stability of chitosan/HA nanoparticles prepared via direct complexation upon dialysis against water: size distribution (A), size and surface charge measured after storage for up to two weeks (B); effect of dilution in different buffers (C).

SI2.2. Atomic Force Microscopy (AFM)

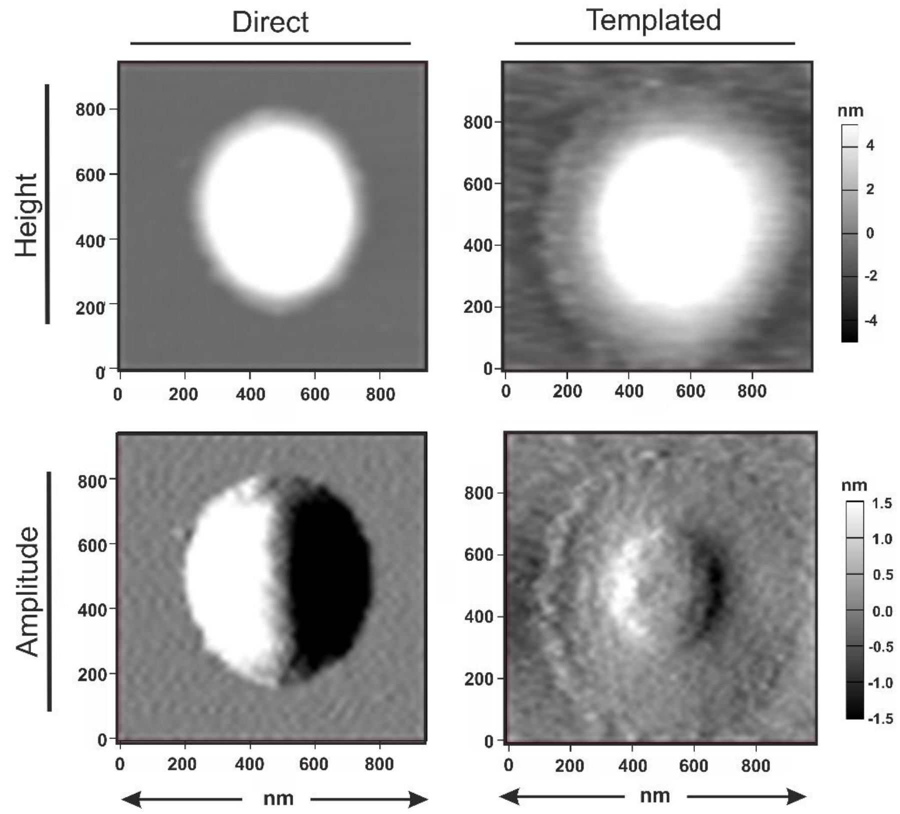

Figure S2: Height and Amplitude images of Chit$_{35}$/HA nanoparticles (obtained through different preparative methods) deposited on a mica substrate. Please note the absence of the HA corona in the nanoparticles prepared via direct complexation with respect to those prepared via the templated method.

SI2.2. Field Flow Fractionation (FFF)

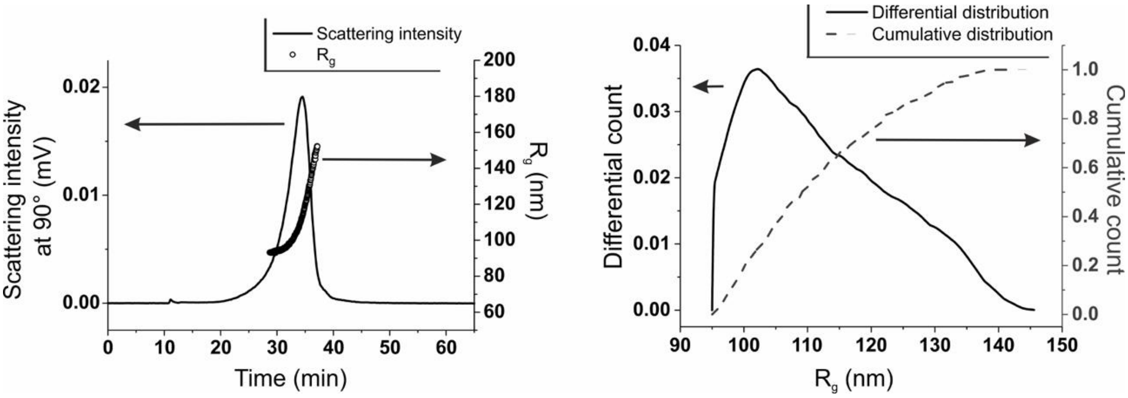

Figure S3: *Left:* representative A4F elugram, obtained using static light scattering detection on templated $Chit_{35}$/HA nanoparticles. *Right:* corresponding differential and cumulative R_g distribution plots.

SI3. Biological studies

Total CD44 expression analysis (flow cytometry, direct staining)

Approximately 1 × 106 RAW264.7 or HCT-116 cells were harvested by gentle scraping in 100 µl FACS (Fluorescence-activated cell sorting) buffer (phosphate buffer solution (PBS) supplemented with 5% (v/v) FBS and 0.1% (m/v) NaN3) per sample tube (1.5 ml Eppendorf tubes) and stained for 30 min at room temperature with Alexa Fluor® 594 anti-mouse/human CD44 Antibody, Clone IM7 (#103054, BioLegend, Cambridge, UK) as per manufacturer's instructions (< 0.25 µg antibody per million cells). To wash off any excess antibody, two serial steps of centrifugation were used and samples were suspended in a final volume of 400 µl PBS. The expression of CD44 was assessed on 10,000 live, individual cells using a BD LSRFortessa cytometer (BD Bioscience, San Jose CA, USA) equipped with the FACSDiva software (v8.0.1). Data were analyzed with FlowJo (vX.0.7, Tree Star, Ashland, OR, USA) after gating live cells in the FSC/SSC window and singlets in the FSC-H/FSC-A window, respectively. The median fluorescence intensity (MFI) of the isotype control (IgG2b, κ) was used as threshold to calculate the MFI of the marker of interest and number of positive cells.

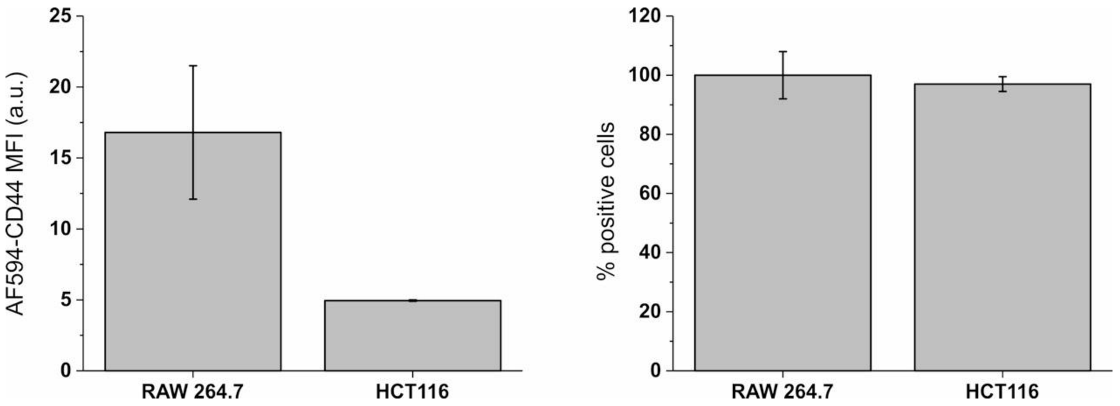

Figure S4: RAW 264.7 and HCT116 cells were analyzed for the expression of total CD44 using an anti-mouse/human CD44 antibody (Clone IM7) labelled with AlexaFluor594. Results are presented as average ± SD (*n* = 3).

References

[1] Rios de la Rosa, J. M.; Tirella, A.; Gennari, A.; Stratford, I. J.; Tirelli, N. *Adv. Healthcare Mater.* **2017,** *6*, 1601012. doi:10.1002/adhm.201601012
